# Drying Kinetics of Microwave-Assisted Drying of Leaching Residues from Hydrometallurgy of Zinc

**DOI:** 10.3390/ma16165546

**Published:** 2023-08-09

**Authors:** Chunlan Tian, Ju Zhou, Chunxiao Ren, Mamdouh Omran, Fan Zhang, Ju Tang

**Affiliations:** 1 Kunming Key Laboratory of Energy Materials Chemistry, Yunnan Minzu University, Kunming 650500, China; tianchunlan@ymu.edu.cn (C.T.); zhouju@ymu.edu.cn (J.Z.); renchunxiao@ymu.edu.cn (C.R.); tangju@ymu.edu.cn (J.T.); 2 Faculty of Technology, University of Oulu, 90570 Oulu, Finland

**Keywords:** zinc hydrometallurgy, residues from leaching stage, microwave drying, drying rate, kinetics model

## Abstract

In the hydrometallurgical process of zinc production, the residue from the leaching stage is an important intermediate product and is treated in a Waelz kiln to recover valuable metals. To ensure optimal results during the Waelz kiln process, it is necessary to pre-treat the residues by drying them first due to their higher water content. This work studies the residue’s drying process using microwave technology. The study results indicate that microwave technology better removes the residue’s oxygen functional groups and moisture. The dehydration process’s effective diffusion coefficient increases as the microwave’s heating power, the initial moisture content, and the initial mass increase. The Page model is appropriate for imitating the drying process, and the activation energy of the drying process for the residues is −13.11217 g/W. These results indicate that microwave technology efficiently dries the residues from the leaching stage. Furthermore, this study provides a theoretical basis and experimental data for the industrial application of microwave drying.

## 1. Introduction

Zinc is highly utilized in various steel, machinery, and chemical industries. It is the third most produced and consumed non-ferrous metal [1,2]. For now, the conventional zinc recovery process is the “roasting—leaching—electrowinning” process, and approximately 80% of total world production comes from this process [3]. The residue from the leaching stage is an essential intermediate product and contains about 30% Zn and 30% Fe. In the zinc recovery process, the residue is primarily subjected to pyrometallurgical treatment and treated in a Waelz kiln to recover valuable metals like zinc, lead, indium, and germanium [4,5]. Therefore, the high moisture content of the residue will lead to difficulties in raising the kiln temperature and the formation of agglomerates in the pre-heat zone, resulting in a lower metal recovery rate. In addition, The dust removal efficiency of the following section and the service life of the dust collection bags will be adversely affected by the high moisture level in the residue [6]. So, the residue needs to be pre-treated by drying before the Waelz kiln process.

Solar irradiation and hot air are usually used to dry materials [7]. Earth receives over 10,000 times more solar energy than the world needs [8,9]. This makes solar drying a sustainable and cost-effective solution for drying various materials and products. However, it is subject to several limitations, such as deterioration of material quality caused by external natural factors and uncontrollable external parameters of the drying process, such as temperature, water content, drying air flow rate, and low drying efficiency [10,11]. Hot air-drying is commonly used in food processing, agriculture, and manufacturing industries to preserve and dry products [12,13,14]. It involves the circulation of hot air around the material, which causes the moisture to evaporate and be carried away. Yang et al. [15] studied drying shiitake mushrooms using hot air. The results indicated that the hot air-drying process enhances the lignification of shiitake mushrooms by increasing lignin, affecting the nutritional and edible properties. Hot air drying can effectively reduce the moisture in the material. However, it is an energy-intensive and time-consuming process.

Microwave is an electromagnetic wave with a frequency between 300 MHz and 300 GHz, a wavelength between 1 mm and 1 m, and a penetrating nature [16]. The materials harvest electromagnetic energy and convert it into thermal energy to remove moisture in the microwave field [17]. Microwave drying is a new method with quick heat and higher thermal efficiency. The principle feature of microwave drying is completely different from other traditional heating methods [18]. The materials are uneven heating via thermal radiation in conventional drying methods. The surface of materials first absorbs the thermal energy, and the internal temperature of the materials increases slowly [19]. However, moisture evaporation mainly occurs on the material’s surface, and the direction of the moisture gradient is from inside to outside. The direction of diffusion of moisture in the materials is directly opposite to the temperature gradient, resulting in blocked diffusion, extended drying time, and reduced drying efficiency. In the microwave drying process, the material is heated evenly. The temperature gradient has the same direction as the moisture gradient, resulting in the same direction of heat and mass transfer, forming an internal pressure gradient, prompting the rapid evaporation of internal moisture, thus allowing the moisture to diffuse to the surface and evaporate quickly, significantly reducing drying time and improving drying effectiveness [20]. Carvalho et al. [21] showed that conventional convection drying (50–70 °C) of malt takes 540–840 min, and the microwave reduces the processing time by about 95%. De Faria et al. [22] investigated the impact of microwave-assisted drying on maize seeds with a wet basis moisture content of 20%, using temperatures of 40, 50, and 60 °C, and power ratings of 0, 0.6, and 1.2 W/g. The results showed that at a temperature of 40 °C and a power rating of 0.6 W/g, the drying time was reduced by approximately 5 h. Demiray Seker et al. [23] also found a significant difference in the drying time of microwave-dried onion flakes compared to convection drying. Convection drying was carried out at different temperatures (50, 60 and 70 °C), 570 min at 50 °C, 360 min at 60 °C and 210 min at 70 °C. Microwave drying was performed at three different microwave power levels, 328, 447 and 557 W. Drying was performed for 66 min at 328 W, 45 min at 451 W and 40 min at 557 W. Compared to traditional drying methods, microwave drying offers advantages such as high energy efficiency, low energy consumption, selective heating, fast heating speed, and absence of by-products [24].

In recent years, a great deal of research has been carried out on microwave drying processes for materials in the food industry, metallurgical industry, etc. Kipcak et al. [25] studied the effect on drying kinetics of microwave power level, rehydration properties, and energy consumption of mussels. The experimental results showed that the trial data were fitted with the Weibull model of relations, and the drying effect was ideal when the microwave power was 360 W. Liu et al. [26] studied the effect of microwaves on the depth drying of Zhaotong lignite. The experimental results showed that the Page model could characterize the microwave depth drying behavior of Zhaotong lignite in diverse settings, and the effective diffusion coefficient enhanced with the power level of the microwave output increases with increasing microwave output power level. Huang et al. [27] have studied the desiccation of ammonium polyvanadate using microwaves and discussed the effects of inert mass, microwave output, and initial moisture content on the drying performance of ammonium polyvanadate. The experimental results showed that the average speed of the drying process was favorably correlated with the output power of the microwaves and that the Modified Page model may accurately designate the process of drying with microwaves of ammonium polyvanadate. Ling et al. [28] studied the microwave drying experiments of zirconium oxide. The experimental results showed that the surface diffusion coefficient increased as the microwave output increased. The sample weight decreased, and the Henderson and Pabis model fit the dehydration process well. Zheng et al. [29] studied natural titanium dioxide’s microwave heating properties and kinetics. They found that the particle size of the material had a considerable impact on the drying effect, leading to the observation that microwaves can fragment mineral particles.

Based on the previous research, this study proposed a solution to the problem of using the microwave to dry zinc-leaching residue for the traditional zinc recovery process with high energy consumption and low efficiency, which realised the solution to the problem of high energy consumption and low efficiency, and effectively removed the water in the leaching residue. The study examines the influence of various microwave heating powers, initial zinc-leaching residue moisture contents, and initial masses on the microwave drying characteristics of zinc-leaching residue. Four classical drying kinetic models, namely Page, Lewis, Wang and Singh, Quadratic, were utilized to fit the experimental information, and the best-fitting model was selected. The effective diffusion coefficient and activation energy were also calculated. The results of this study provide valuable research evidence and reference for the discipline of acid-leaching residue drying.

## 2. Experimental Section

### 2.1. Experimental Substances

The zinc leaching residue selected for this experiment was collected from a zinc smelter in Yunnan Province, China; the dry basis moisture of the slag is in the region of 9.2%, and heated in a tumble dryer at 60 °C for 24 h to maintain a constant composition. Sulphuric acid is used to recover valuable metals in hydrometallurgical zinc production. In this paper, the leaching residue from an acid leaching stage. The acid residue underwent analysis using the method recommended by the national standard of the People’s Republic of China (GB/T 10561-2019) [30], and the main element contents are shown in Table 1.

**Figure 1:**
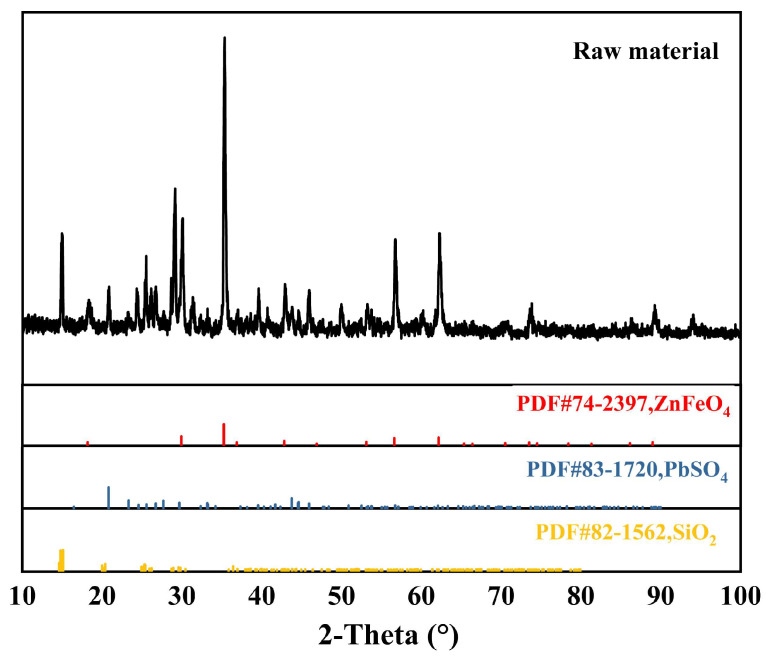
XRD results of acid leaching residue.

X-ray diffraction (XRD) was performed to analyze the material composition of the residue. The outcomes, as shown in Figure 1, indicate that the primary components of the original material are lead sulfate and zinc ferrite. The copper element is below 5%, and no associated phase is found in the XRD analysis.

**Figure 2:**
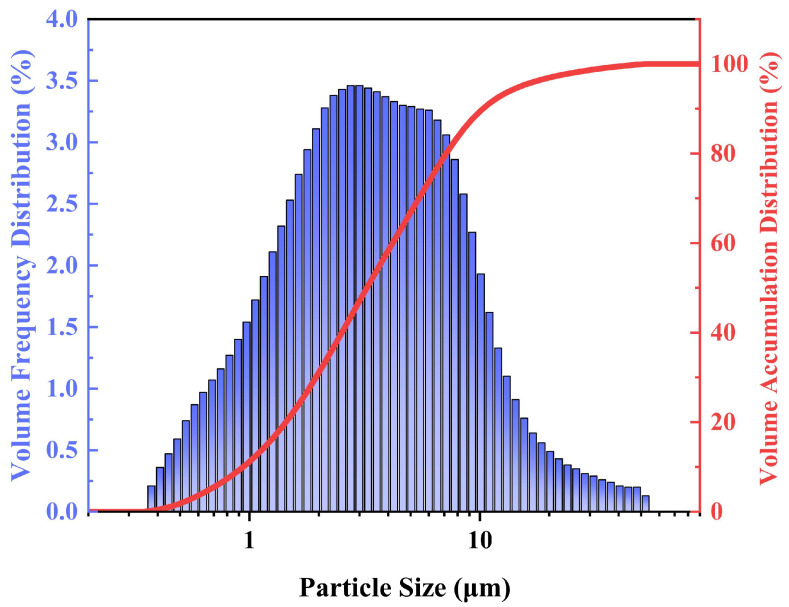
Distribution of particle size of acid leaching residue.

The size and distribution of the particles of the acid-leaching residue were analyzed using a laser particle sizer. Figure 2 presents the histogram of the size distribution and the accumulated volume distribution curve of the acid-leaching resin. As can be seen from Table 2, the nominal particle size of the acid wash in the region of 0.938 to 15 µm with a volume mean diameter (Mz) of 4.19 µm and a median particle size (D_50_) of 3.23 µm.

### 2.2. Experimental Equipment

**Figure 3:**
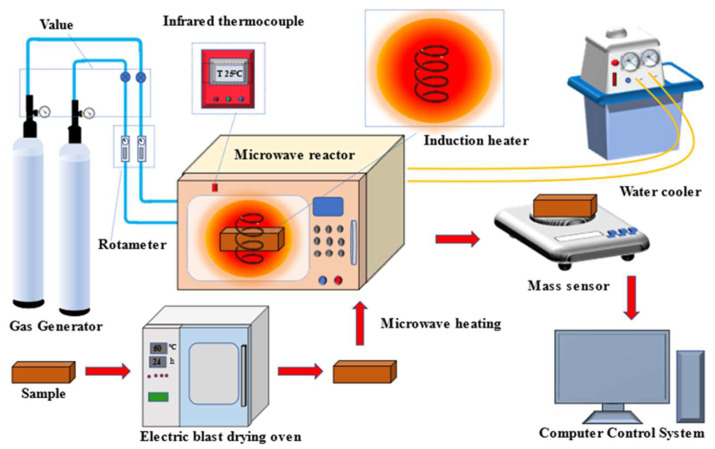
Diagram of microwave drying equipment.

A diagram of the microwave drying plant is shown in Figure 3. The plant comprises three primary components: the Gas delivery unit, the heating installation, and the information-gathering tool. The gas supply system regulates the gas atmosphere in the reaction chamber during drying. The heating system is a microwave drying oven that heats the sample to dry it, while the data acquisition system comprises a mass sensor and a computer steering gear. The mass sensor detects changes in the material’s quality during the microwave heating and drying process, and the computer control system records the data to track changes in quality.

### 2.3. Experimental Procedures

The zinc-leaching residue from a factory was used as the sample for the experiment. To guarantee the validity of the scores, a specific amount of the residue was mixed with water and placed in a rectangular corundum crucible measuring 9 cm × 6 cm × 1.6 cm. This was done to eliminate any influence that the starting condition of the specimen may have on the microwave drying process during the experiment. The crucible was then placed in a microwave-drying oven for the experiment. This study aimed to investigate the impact of microwave heating power, initial moisture content, and initial mass on the microwave drying of the residue. The experiment involved varying the microwave heating power (160 W, 320 W, 480 W, 640 W, 800 W), initial moisture content (3.25%, 6.55%, 8.15%, 9.75%, 11.35%), and initial mass (10 g, 15 g, 20 g, 25 g, 30 g) of the residue.

Zinc leachates with 8.60% initial moisture and 20 g mass were selected by the controlled variable method and dried at variables in microwave heating power (160 W, 320 W, 480 W, 640 W, 800 W); test samples were made up using the concept of dry basis moisture percent. Zinc leachate Samples with various initial moisture contents (3.25%, 6.55%, 8.15%, 9.75%, 11.35%) were prepared, weighed 20 g, and dried at 480 W microwave heating power; zinc leachate with an initial moisture content of 8.60% and initial masses of 10 g, 15 g, 20 g, 25 g, and 30 g were selected using the controlled variable method. The three single-variable trials were carried out at a heating power of 480 W. The sample masses were recorded at 30 s intervals for all three univariate experiments until the sample masses did not change on three occasions.

## 3. Methods

### 3.1. Calculation of the Relevant Parameters

(1) Moisture content

The calculation of the moisture content (*M_t_*) of acid-leaching residue at a given time (*t*) during the experiment is based on the principle of mass conservation. This is done using the Equation (1) provided.

(1) $$M_{t}=\left[1-\frac{m_{0}\times \left(1-M_{0}\right)}{m_{t}}\right]\times 100\%\;$$

where: *M_t_*—the water content of the acid leaching residue at time *t*, %; *M*_0_—initial moisture content of the zinc leaching residue, %; *m*_0_—the initial mass of the acid leaching residue (with water), g; *m_t_*—the mass of the acid leaching residue at time *t*, g.

(2) Hydration ratios

(2) $$MR=\frac{M_{t}-M_{e}}{M_{0}-M_{e}}\times 100\%\;$$

where, *M_t_*—the water holding capacity of acid leach residue at time *t*, %; *M_e_*—equilibrium wet basis moisture content, %; *M*_0_—the initial water content of acid leaching residue, %.

(3) Instantaneous drying rate

The effect of water removal from the acid-leaching residue at time *t* can be expressed in terms of the instantaneous drying rate, which is calculated according to Equation (3):

(3) $$R=-\frac{M_{\left(t+\Delta t\right)}-M_{t}}{\Delta t}\;$$

where *R*—instantaneous drying rate, g/s; *M_t_*—mass of acid leaching residue from drying to *t*, g; *M*_(*t*+Δ*t*)_—mass of acid leaching residue from drying to (*t* + Δ*t*), g.

(4) Average drying rate

(4) $$Ra=-\frac{m-m_{0}}{t}\;$$

where, *Ra*—average drying rate, g/s; *m*—mass of acid leaching residue at the end of drying, g; *m*_0_—initial mass of acid leaching residue (with moisture), g; *t*—time used at the end of drying, s.

### 3.2. Numerical and Kinetic Models for Thin Layer Drying

The thin-layer drying method involves transferring heat from hot air to a wet material by means of convection. The heated air is then forced to escape through the thin layer of the substance, carrying out the evaporated water vapor [31]. Currently, The primary focus of drying kinetics research is the use of mathematical simulations of thin-layer drying curves to derive thin-layer drying models. Several thin-layer drying models are available, generally categorized as theoretical, semi-theoretical, empirical, and semi-empirical. Empirical models share similarities, given that they rely heavily on laboratory testing conditions and provide limited insight into the drying behavior of the material [32]. Empirical models are developed using experimental data and measurement analysis and can be used to simulate drying processes [33]. Theoretical models consider external conditions and the influence of moisture transport within the material [34]. Semi-theoretical and empirical models which consider only the resistivity of the external resistance to moisture exchange with air can give more accurate results., improve predictions of drying behavior, and reduce assumptions by relying on experimental data [35]. In this work, Four common drying kinetic models, Page, Lewis, Wang and Singh, Quadratic, as shown in Table 3, were selected to fit the experimental data during the microwave drying of zinc leaching residue. To choose the most appropriate drying model, a series of statistical indicators were used, including the fit coefficient (R^2^), residual sum of squares (RSS), and F-value values. The expressions for these statistical indicators are shown in Table 4.

## 4. Results and Discussion

### 4.1. Effect of Microwave Heating Power on the Drying of Acid-Leaching Residues Using Microwaves

**Figure 4:**
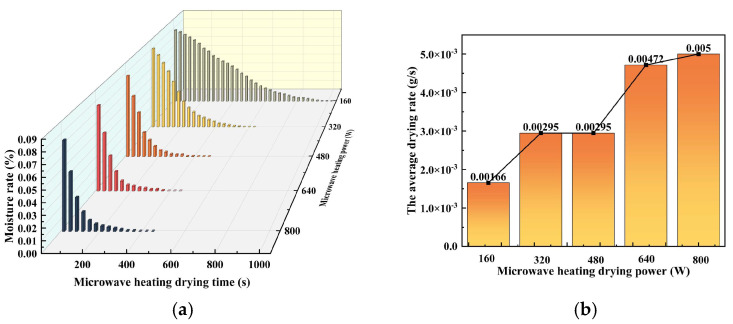
(**a**) Moisture rate-microwave heating time curves of acid leaching residue of zinc at different microwave heating powers; (**b**) Average drying rate-microwave heating power curves of acid leaching residue of zinc at different microwave heating powers.

Figure 4 illustrates the moisture content variation curves of the acid-leaching residue with microwave heating time and the variation curves of the average drying rate with the microwave heating power at different levels. The graph shows that below the 160 W, 320 W, 480 W, 640 W, and 800 W microwave heating power, the drying completion time is 960 s, 570 s, 420 s, 390 s, and 390 s, respectively. Drying time gradually decreases as microwave power increases. The acid-leaching residue microwave drying process can be broken down into three stages. The first stage consists of preheating, which involves using microwave heating to evaporate the internal free water in the sample. During this stage, there is a small amount of evaporation, resulting in a minimal change in the moisture content. The second stage is steaming, when the internal bound water evaporates from the sample’s surface. The rate of decline in moisture content is gradually accelerated during this stage. The end of evaporation is the third stage, during which only a small amount of bound water is left in the sample. The moisture content shows a slow decline, eventually reaching zero. The study reveals that the removal rate depends on the initial moisture content, mass, and microwave heating power. The higher the microwave heating power, the more microwaves are absorbed by the acid-leaching residue, resulting in an accelerated drying rate [36].

Figure 4b plots the average drying rate for the acid leach residue at different microwave heating powers. At 160 W, 320 W, 480 W, 640 W, and 800 W, the average drying rates were 0.00166 g/s, 0.00295 g/s, 0.00295 g/s, 0.00472 g/s, and 0.005 g/s. Interestingly, the average drying rate was the same at 320 W and 480 W, and it accelerated above 480 W. Insufficient penetration of the microwaves into the acid-leaching residue could be the reason for the lower average drying rate at low microwave heating power [37]. If the microwave heating power exceeds 480 W, the average drying rate of acid-leaching residue increases slowly. This could be attributed to the saturation of microwave absorption by the residue. Further, an increase in microwave heating power causes the acid leach residue to no longer absorb microwaves, resulting in a wastage of microwave energy as the microwaves pass through the residue [38].

### 4.2. Effect of Initial Moisture Content on the Drying of Acid-Leaching Residues Using Microwaves

**Figure 5:**
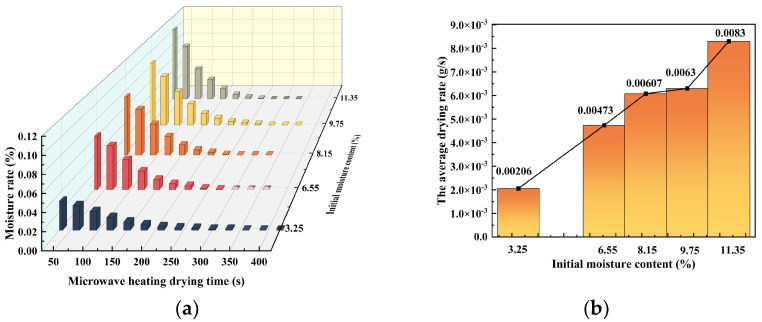
(**a**) Moisture rate-time curves for different initial moisture contents of the material. (**b**) Average drying rate-initial moisture content curve for different initial moisture contents of the material.

Figure 5 displays the moisture content variation curves of the acid leaching residue concerning the change of microwave exposure time and average drying rate variation curves with modification of initial moisture content at different initial moisture contents. The acid-leaching residue’s drying completion time was 360 s, 300 s, and 270 s, 300 s, 270 s, as the initial moisture content increased, as shown in Figure 5a. The results in Figure 5b demonstrate that as the initial moisture content of the acid-leaching residue increased from 3.25% to 11.35%, the average drying rate also increased. This can be interpreted as being due to more water molecules in the residue at a higher initial moisture content, which absorbs more microwave energy and reaches boiling point more quickly. According to the study, the higher moisture content in acid-leaching residue leads to more absorption of microwaves and subsequent boiling of water molecules which escape into the environment.

### 4.3. Effect of Initial Mass on the Drying of Acid-Leaching Residues Using Microwaves

**Figure 6:**
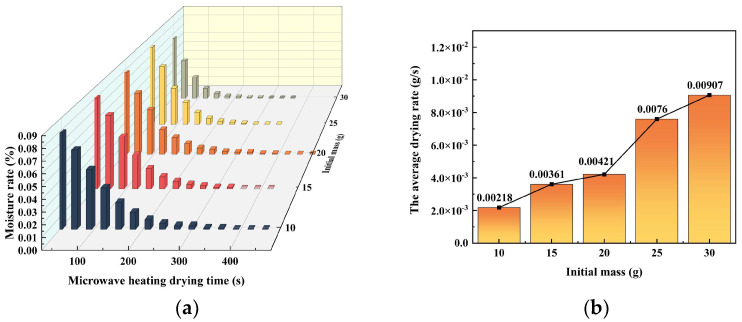
(**a**) Moisture rate-microwave heating drying time curves for acid leaching residue at different initial masses; (**b**) Acid leaching residue at different initial mass—average drying rate curve.

This study examined the drying behavior of acid-leaching residues with varying initial masses. The residues were dehydrated using a microwave heating power of 480 W, and the corresponding changes in the moisture content and the rate of drying were recorded. The results are shown in Figure 6a, where the average drying rates and completion times varied depending on the initial mass of the residues. The drying rates ranged from 0.00218 g/s to 0.00907 g/s, and the completion times ranged from 300 s to 420 s.

The study found that the proportion of drying was the highest, and the completion time was the shortest when the initial mass of the acid-leaching residue was 25 g. Additionally, it can be seen from Figure 6b that the average rate of drying tended to increase in proportion to the increase in the initial mass of the acid leach residue.

### 4.4. Results and Analysis of the X-ray Diffractometer

**Figure 7:**
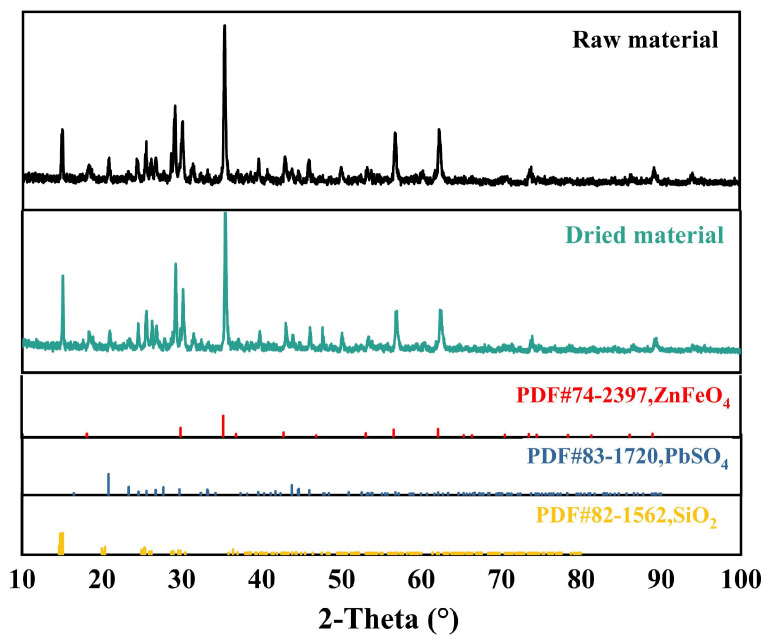
XRD spectra of acid-leaching residue before and after drying.

The phase composition of the Zn leach residue was essentially the same before and after microwave drying according to XRD tests on the Zn leach residue and after drying, indicating that microwave drying does not affect the phase composition of the material. The XRD pattern of zinc leaching slag before and after drying is shown in Figure 7.

### 4.5. Results and Analysis of the Fourier Transform Infrared Spectroscopy

To examine the impact of microwave heating applied to the microscopic functional groups on the surface of samples of zinc leaching residue, FT-IR spectroscopy (NICOLET-IS10, Nicolet, USA) was conducted on both dried and undried samples within the spectral range of 4000~450 cm^−1^ was covered.

**Figure 8:**
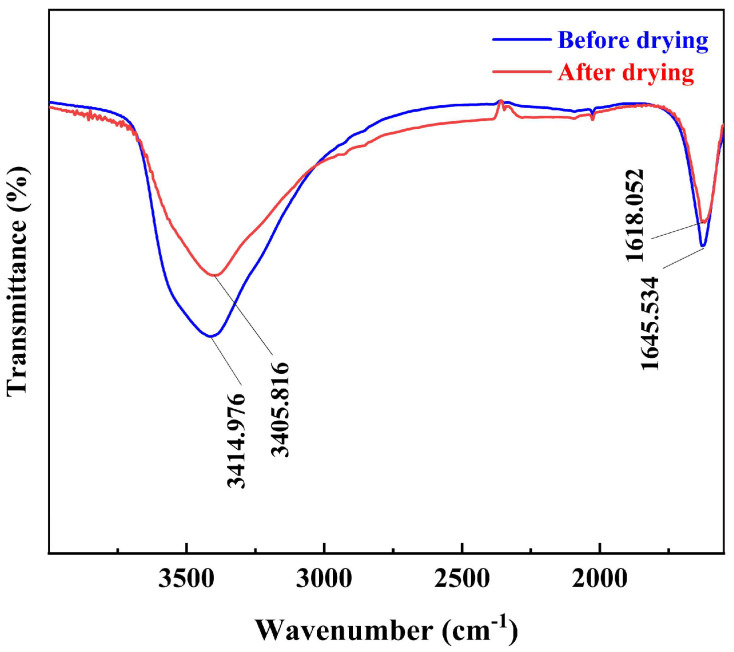
FT-IR spectra of acid-leaching residue before and after drying.

Water’s absorption peaks and molecular bending-stretching vibrational frequencies appear around 1640 cm^−1^ and 3400 cm^−1^, respectively [39]. As depicted in Figure 8, the FT-IR spectra of the pre-dried acid leaching residue showed a distinctive absorbance peak at 3414.976 cm^−1^, which is indicative of stretching vibrations of the O-H bond and at 1645.534 cm^−1^ due to bending vibrations within the H-O-H plane. The FT-IR spectra taken from the dried acid leaching residue showed a characteristic absorcent peak at 3405.816 cm^−1^ arising from the extension vibration of the O-H bond and a characteristic absorcent peak at 1618.052 cm^−1^ arising from the inflexion vibration in the H-O-H plane. In contrast, it was found that the characteristic absorption peak due to the extension vibration of the O-H bond was weaker in the dried acid-leaching residue than in the acid-leaching residue before drying, indicating that the microwave was effective in removing the oxygen-containing functional groups from within the acid leaching residue. This experimental result is also in accordance with the work by Lin et al. [40].

### 4.6. Drying Kinetics Model Fitting Process

**Figure 9:**
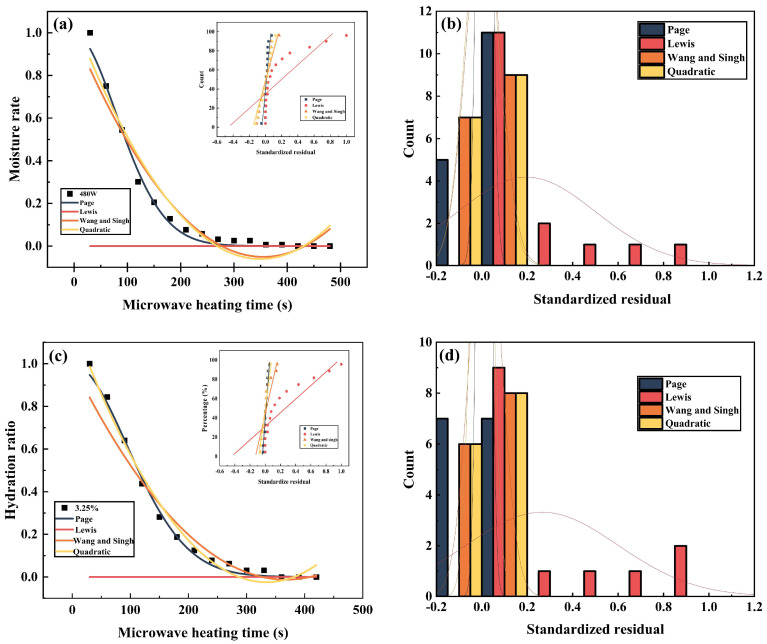

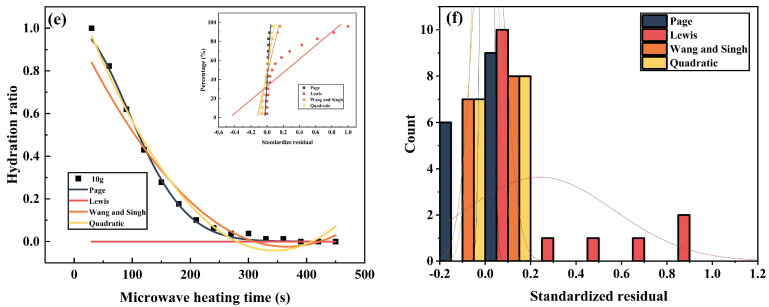
(**a**,**c**,**e**) Fitted different mathematical models to empirical data derived from the microwave drying process of zinc leach residues; (**a**) microwave heating power of 480 W, controlled water content of 8.60%, and mass of zinc leaching residue of 20 g; (**c**) moisture content of 3.25%, controlled microwave heating power of 480 W and mass of zinc leaching residue of 20 g; (**e**) microwave heating power of 480 W, controlled water content of 8.60% and mass of zinc leaching residue of 10 g. (**b**,**d**,**f**) shows the residual histograms for Page, Lewis, Wang and Singh, Quadratic models fitted to the hydration ratio data model: (**b**) residual histograms for Page, Lewis, Wang and Singh, Quadratic models fitted to a microwave heating power of 480 W, a control moisture content of 8.60% and a zinc leaching residue mass of 20 g; (**d**) residual histograms for Page, Lewis, Wang and Singh, Quadratic models fitted to a zinc leaching residue moisture content of 3.25%, a controlled microwave heating power of 480 W and a mass of 20 g. (**f**) residual histograms for Page, Lewis, Wang and Singh, Quadratic models fitted to a zinc leaching residue mass of 10 g, a controlled microwave heating power of 480 W and a moisture content of 8.60%.

The experimental results of microwave drying of leaching residue obtained for different experimental conditions were fitted using Page, Lewis, Wang and Singh Quadratic models. Table 5, Table 6 and Table 7 and Figure 9 display the fitted data for the models. Page, Lweis, Wang and Singh’s quadratic model was used for microwave heating power of 480 W, an initial moisture content of 3.25%, and an initial mass of 10 g. In addition, the models were used to fit hydration ratio curves and experimental data with varying microwave heating powers, initial moisture content, and initial mass. A normal distribution plot was utilized as a reference for comparison [41]. Von the four models, the Page model was deemed to be the one which best fitted the experimental data.

The Page model was used to fit the zinc leachate moisture content data at different microwave heating powers (160 W, 320 W, 480 W, 640 W, 800 W), initial moisture contents (3.25%, 6.55%, 8.15%, 9.75%, 11.35%) and initial masses (10 g, 15 g, 20 g, 25 g, 30 g). As shown in Table 8, the results showed that the Page model had higher R^2^ values, smaller RSS values, larger F-values, lower Chi-sqr values and better normal distribution than the other three drying fit models. These results indicate that the Page model is the most suitable model to describe the drying kinetics of zinc leachate compared to the other three models.

**Figure 10:**
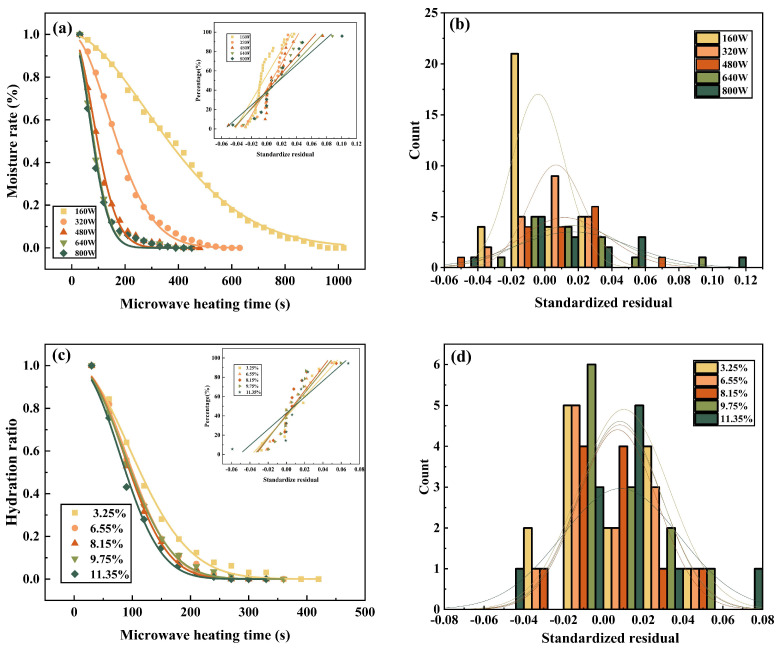

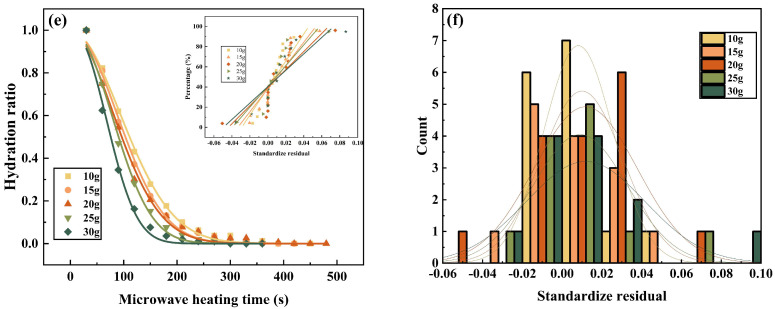
(**a**,**c**,**e**) Adaptation by Page hydration ratio data for variable microwave heating power, initial moisture content and initial mass, with small plots representing normal probability plots: (**a**) Fitted of Page hydration ratio data for different microwave heating power (160 W, 320 W, 480 W, 640 W, 800 W); (**c**) Fitted of Page hydration ratio data for different initial moisture content (3.25%, 6.55%, 8.15%, 9.75%, 11.35%); (**e**) Fitted graphs of Page hydration ratio data for different initial masses (10 g, 15 g, 20 g, 25 g, 30 g). (**b**,**d**,**f**) Histograms of residuals under the Page hydration ratio data model with different microwave heating power, initial moisture content and initial mass: (**b**) Histograms of residuals under the Page hydration ratio data model with different microwave heating power (160 W, 320 W, 480 W, 640 W, 800 W); (**d**) Histograms of residuals under the Page hydration ratio data model with different initial moisture content (3.25%, 6.55%, 8.15%, 9.75%, 11.35%); (**f**) histograms of residuals under the Page hydration ratio data model for different initial masses of 10 g, 15 g, 20 g, 25 g, 30 g).

Based on the observations in Figure 10a,c,e, the hydration rate data at different microwave heating powers, moisture contents, and masses can be fitted well with the Page model. Also, as shown in Figure 10b,d,f, the hydration rate data under the same conditions follow a normal distribution. These results also suggest that the Page model can effectively explain the behavior of microwave-dried zinc leachates under different conditions [42].

### 4.7. Calculating the Diffusion Coefficient and Activation Energy

**Figure 11:**
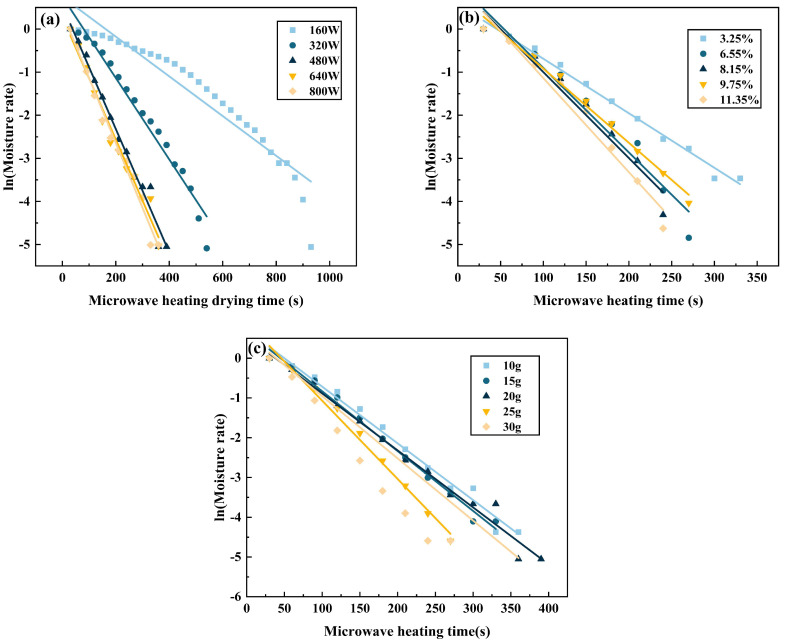
Linear fit of *ln MR* versus *t* for different.(**a**) Linear fit of *ln MR* versus *t* for various microwave heating powers; (**b**) Linear fit of *ln MR* versus *t* for initial different moisture contents; (**c**) Linear fit of *ln MR* versus *t* for several initial masses.

Fick’s second law of diffusion is frequently used to describe the drying process involved in various materials [43]. A longitudinal fit of *ln MR* versus *t* was performed using the obtained microwave drying data, as shown in Figure 11. This experiment assesses the surface diffusion coefficient, obtaining the steepness and intercept from the linear plot formed between *ln MR* and *t*. The diffusion out of water in the zinc leach residue over the drying process is described by Fick’s second law of diffusion [44].

(5) $$MR=\frac{6}{\pi^{2}}\sum_{n=1}^{n=\infty }\frac{1}{n^{2}}\;exp\left[-\frac{4n^{2}\pi^{2}D_{e}}{d^{2}}t\right]\;$$

During a long drying time, *n* = 1, Equation (5) above can be simplified as

(6) $$MR=\frac{6}{\pi^{2}}\;exp\left[-\frac{4\pi^{2}D_{e}}{d^{2}}t\right]$$

Taking the reciprocals to the logarithm of both sides of Equation (6) simultaneously gives Equation (7):

(7) $$\ln MR=\ln\frac{6}{\pi^{2}}-\frac{4\pi^{2}D_{e}}{d^{2}}t$$

From Equation (7), *D_e_* represents the effective diffusion coefficient (m^2^/s), *d* refers to the particle size of the material (m), and *t* denotes the drying time (s).

Suitably, by fitting Equation (7) to relevant empirical data, a slant a of the fitted line can be derived, and the average effective diffusion coefficient may be estimated from Equation (7) using this slant:

(8) $$D_{e}=-a\;\frac{d^{2}}{\pi^{2}}\;$$

**Figure 12:**
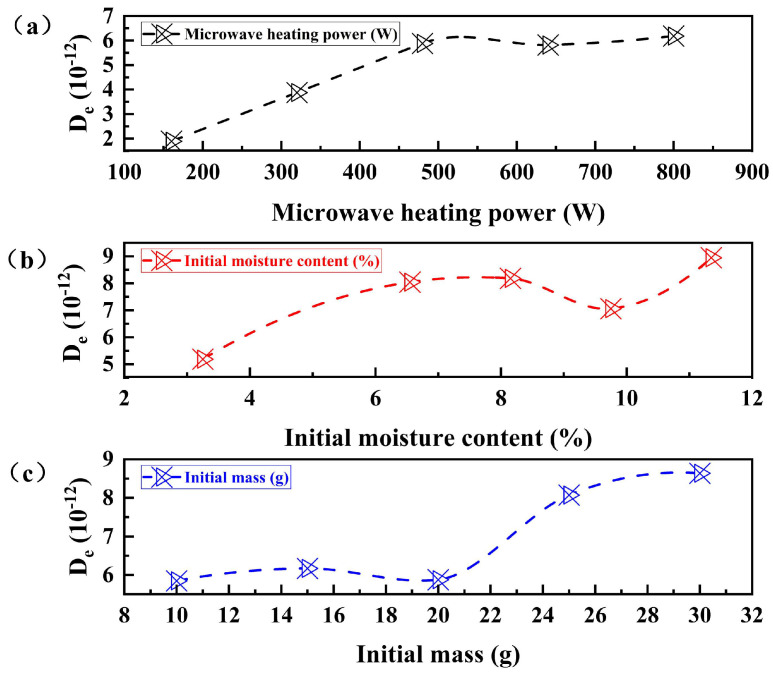
Effect of different microwave heating power, initial moisture content and initial mass on the effective diffusion coefficient of acid leaching residue. (**a**) The effect of different microwave heating power on the effective diffusion coefficient of acid-leaching residue; (**b**) The effect of different initial water content on the effective diffusion coefficient of acid-leaching residue; (**c**) the Influence of different initial mass on the effective diffusion coefficient of acid-leaching residue.

Table 9 shows the effective diffusion coefficients of acid leaching residue for different microwave heating powers, initial moisture content, and initial mass. Figure 12 shows the trend of the effect of different microwave heating power, initial moisture content and initial mass on the effective diffusion coefficient of acid leaching residue. Specifically, adjusting the microwave power to 800 W, the initial moisture content is 11.35%, and the initial mass is 30 g, the diffusion coefficient is the highest. If the microwave heating power is increased, a corresponding increase in the diffusion coefficient of the zinc leach residue is observed. This is because the faster heating rate leads to a more significant diffusion coefficient of the residue [45].

Based on the relationship between hydration ratio and heating time, it was found that the equilibrium time of the hydration ratio is higher for a microwave heating power of 640 W than that of 480 W. The reduction in the diffusion coefficient of zinc leaching residue could be attributed to fitting errors. The diffusion coefficient in zinc leaching residue increases gradually as the moisture content increases, but only up to a certain point. When the moisture content reaches 8.15%, the diffusion coefficient begins to weaken due to the increase in water. This weakening is caused by the water molecules inside the residue becoming weaker. However, when the moisture content is 11.35%, there may be a fitting error that causes an increase in the diffusion coefficient. The efficiency of zinc leaching residue to absorb microwaves and its diffusion coefficient increases gradually as its initial mass increases. This is because a small mass of less than 20 g cannot absorb enough energy, resulting in a low diffusion coefficient [46].

Dadali et al. proposed an alternative method to estimate the activation energy for microwave drying [47]. Activation energy is the energy needed to convert a molecule from its resting state to an active state where chemical reactions can occur. *D_e_* depends on the material mass and the microwave heating power intensity for the Arrhenius-type equation:

$$D_{e}=D_{o}\exp\left(\frac{E_{a}m}{P_{m}}\right)$$

where *D_e_* is a function of material quality and microwave power level; *D*_0_ is the finger front factor of the Arrhenius equation in m^2^/s, *E_a_* which is the activation energy (W/g), *m* which is the mass of the product 20 g and *P_m_* which gives the microwave heating power (W).

The calculation equation can be described as,

(9) $$\ln D_{e}=\ln D_{0}+\frac{20E_{a}}{P_{m}}$$

**Figure 13:**
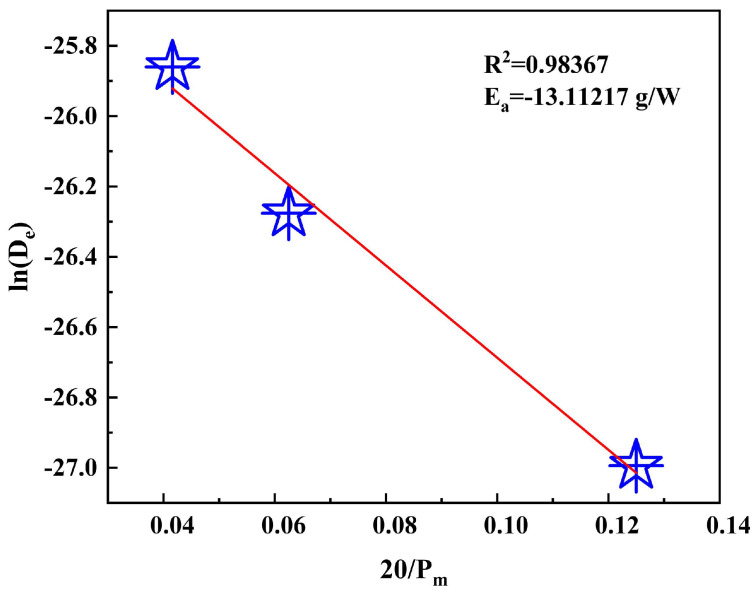
Calculating the activation energy needed for microwave drying.

As can be seen in Figure 13, the diffusion coefficient increases linearly over the microwave power range extending from 160 to 480 W. As a result, three different microwave heating powers were selected, namely 160 W, 320 W, and 480 W. Using the equivalence formula combining *ln D_e_* and 20/*P_m_*, the activation energy was determined to be −13.11217 g/W.

## 5. Conclusions

This study examines the importance of microwave heating power, initial moisture content, and initial mass concerning wet zinc leaching residue drying.

The microwave drying of zinc-leaching residue is affected by various factors, including microwave heating power, initial moisture content, and initial mass. As these factors increase, the average drying speed also increases. This can be attributed to the fact that a larger mass of zinc-leaching residue absorbs more microwave energy when other conditions remain constant, resulting in a faster temperature rise and, subsequently, a quicker drying performance. The higher the microwave heating power, the faster the heating rate and the greater the microwave radiation on the leakage residue surface. This results in a step improvement in the microwave drying frequency.

Additionally, a relatively high initial moisture content signifies more water present, resulting in more water molecules absorbing microwaves. This causes additional water molecules to be at the boiling point and released through the exposed surface of the leach residue. The phase of leaching residue did not change before and after drying. Microwave technology effectively removes oxygen-containing functional groups in the leaching residue and reduces its moisture content.

This study used the quadratic model of Page, Lewis, Wang and Singh to fit the zinc leach slag hydration ratio data. The experiments were conducted at a heating power of 480 W, an initial water content of 8.60%, and an initial mass of 20 g, respectively. The results showed that the Page model was the best-fitting model. The Page model was further used to match the data for different masses, microwave heating power, and moisture content to produce R^2^, RSS, F-values, and reduced Chi-Sqr values under the Page model. As the microwave heating power, initial moisture content, and initial mass increased, the effective diffusion coefficient tended to grow, and the average drying rate gradually increased.

Fick’s second law of diffusion was used to estimate the diffusion coefficient, which showed an overall increase in microwave heating power, initial moisture content, and initial mass. A more significant effective diffusion coefficient indicates a faster reaction rate, indicating that microwave heating can be highly selective in accelerating the reaction rate. The activation energy calculated for microwave-dried leach residues was −13.11217 g/W, based on the relationship between microwave power and activation energy.

## Figures and Tables

**Table 1:** Chemical elements of raw materials for acid leach residue (wt%).

**Element**	**Fe_2_O_3_**	**ZnO**	**SiO_2_**		**PbO**	**CuO**	**MnO**	**K_2_O**
Contain (wt%)	36.448	29.243	14.617		11.262	1.720	0.637	0.479
**Element**	**CdO**	**Tr_2_O_3_**	**TiO_2_**	**C1**	**SrO**	**V_2_O_5_**	**Ag_2_O**	**ZrO_2_**
Contain (wt%)	0.414	0.358	0.289	0.240	0.238	0.084	0.055	0.048

**Table 2:** Particle size distribution of acid leaching residue.

Particle Size Distribution Ratio	D_10_	D_20_	D_30_	D_40_	D_50_	D_60_	D_70_	D_80_	D_90_	D_95_	Mz
Particle size/µm	0.938	1.438	1.943	2.541	3.23	4.18	5.44	7.14	10.3	15	4.19

**Table 3:** The trial information on drying kinetics models by fitting.

No.	Model	Model Expression
1	Page	$M_{R}=exp\left(-kt^{n}\right)$
2	Lewis	$M_{R}=exp\left(-kt\right)$
3	Wang and Singh	$M_{R}=1+at+bt^{2}$
4	Quadratic	$M_{R}=a+bt+ct^{2}$

**Table 4:** Mathematical model statistical indicators.

Statistical Indicators	Equations
slimming coefficient	$R^{2}=\frac{\sum \left(MR_{exp}-{\bar{MR}}_{exp}\right)\left(MR_{pre}-{\bar{MR}}_{pre}\right)}{\sqrt{{\left(MR_{exp}-{\bar{MR}}_{exp}\right)}^{2}{\left(MR_{pre}-{\bar{MR}}_{pre}\right)}^{2}}}$
residual sum of squares	$RSS=\overset{N}{\underset{i=1}{\sum }}{\left(MR_{exp}-MR_{pre}\right)}^{2}$
F-Value	$F\text{-}Value=\frac{MStreatment}{MS_{error}}$

**Table 5:** Data on the heating power of 480 W microwave fitted to various models.

Model	Params	R^2^	RSS	F-Value
Page	k = 1.34012 × 10^−4^ *n* = 1.87167	0.99117	0.01233	1139.59521
Lewis	k = 0.61636	−0.44709	2.02034	1.38423 × 10^−7^
Wang and Singh	a = −0.00592 b = 8.34054 × 10^−6^	0.93963	0.08428	160.80051
Quadratic	a = 1.06336 b = −0.00643 c = 9.20115 × 10^−6^	0.94357	0.07878	108.6863

**Table 6:** Data for different models fitted to acid-leaching residue with a moisture content of 3.25%.

Model	Params	R^2^	RSS	F-Value
Page	k = 8.09485 × 10^−5^ *n* = 1.91271	0.9944	0.00822	1786.59595
Lewis	k= 0.61636	−0.67299	2.45557	9.87034 × 10^−8^
Wang and Singh	a = −0.00551 b = 7.48395 × 10^−6^	0.95554	0.06526	219.75131
Quadratic	a = 1.19557 b = −0.0073 c = 1.09222 × 10^−5^	0.98555	0.02121	375.17481

**Table 7:** Data for different models fitted to an initial mass of 10 g of acid-leaching residue.

Model	Params	R^2^	RSS	F-Value
Page	k = 7.85963 × 10^−5^ *n* = 1.92866	0.9965	0.00529	2907.07712
Lewis	k = 0.61636	−0.56991	2.373341	1.09979 × 10^−7^
Wang and Singh	a = −0.00559 b = 7.63484 × 10^−6^	0.95834	0.06299	238.41688
Quadratic	a = 1.16422 b = −0.007 c = 1.01621 × 10^−5^	0.98082	0.029	306.8248

**Table 8:** Experimental factors and coding.

	Condition	Params	R^2^	RSS	F-Value
Microwave heating power	160 W	k = 2.28837 × 10^−5^ *n* = 1.75036	0.99763	0.00891	15,116.38519
	320 W	k = 5.76718 × 10^−5^ *n* = 1.82929	0.9971	0.00644	5620.87119
	480 W	k = 1.34012 × 10^−4^ *n* = 1.87167	0.99117	0.01233	1139.59521
	640 W	k = 1.07697 × 10^−4^ *n* = 1.98482	0.98746	0.0154	715.01768
	800 W	k = 1.56908 × 10^−4^ *n* = 1.91609	0.98269	0.02051	513.26985
Moisture content	3.25%	k = 8.09485 × 10^−5^ *n* = 1.91271	0.9944	0.00822	1786.59595
	6.55%	k = 3.40842 × 10^−5^ *n* = 2.15056	0.99572	0.00574	1874.06956
	8.15%	k = 3.72183 × 10^−5^ *n* = 2.15197	0.99637	0.00452	2025.6383
	9.75%	k = 5.95382 × 10^−5^ *n* = 2.04029	0.99566	0.00558	1855.28185
	11.35%	k = 5.37631 × 10^−5^ *n* = 2.10929	0.99196	0.00957	870.83348
Mass	10 g	k = 7.85963 × 10^−5^ *n* = 1.92866	0.9965	0.00529	2907.07712
	15 g	k = 6.82384 × 10^−5^ *n* = 1.98976	0.99511	0.00694	1895.00272
	20 g	k = 1.34012 × 10^−4^ *n* = 1.87167	0.99117	0.01233	1139.59521
	25 g	k = 6.04899 × 10^−5^ *n* = 2.07446	0.99423	0.00714	1318.63153
	30 g	k = 5.17039 × 10^−5^ *n* = 2.19605	0.98961	001151	665.16048

**Table 9:** Effective diffusion coefficients of acid leaching residue for different microwave heating powers, initial moisture content, and initial mass.

Microwave Heating Powder (W)	*D_e_* (10^−12^ m^2^/s)	Moisture Content (%)	*D_e_* (10^−12^ m^2^/s)	Mass (g)	*D_e_* (10^−12^ m^2^/s)
160	1.89056	3.25	5.18777	10	5.84803
320	3.87545	6.55	8.04206	15	6.1679
480	5.87673	8.15	8.1815	20	5.87673
640	5.81932	9.75	7.06192	25	8.07487
800	6.18021	11.35	8.94838	30	8.63671

## Data Availability

The data presented in this study are available upon request from the corresponding author. The data are not publicly available due to technical or time limitations.
